# Gefitinib-Induced Killing of NSCLC Cell Lines Expressing Mutant *EGFR* Requires BIM and Can Be Enhanced by BH3 Mimetics

**DOI:** 10.1371/journal.pmed.0040316

**Published:** 2007-10-30

**Authors:** Mark S Cragg, Junya Kuroda, Hamsa Puthalakath, David C. S Huang, Andreas Strasser

**Affiliations:** The Walter and Eliza Hall Institute of Medical Research, Melbourne, Australia; University of California Los Angeles, United States of America

## Abstract

**Background:**

The epidermal growth factor receptor (EGFR) plays a critical role in the control of cellular proliferation, differentiation, and survival. Abnormalities in EGF-EGFR signaling, such as mutations that render the EGFR hyperactive or cause overexpression of the wild-type receptor, have been found in a broad range of cancers, including carcinomas of the lung, breast, and colon. EGFR inhibitors such as gefitinib have proven successful in the treatment of certain cancers, particularly non-small cell lung cancers (NSCLCs) harboring activating mutations within the *EGFR* gene, but the molecular mechanisms leading to tumor regression remain unknown. Therefore, we wished to delineate these mechanisms.

**Methods and Findings:**

We performed biochemical and genetic studies to investigate the mechanisms by which inhibitors of EGFR tyrosine kinase activity, such as gefitinib, inhibit the growth of human NSCLCs. We found that gefitinib triggered intrinsic (also called “mitochondrial”) apoptosis signaling, involving the activation of BAX and mitochondrial release of cytochrome *c*, ultimately unleashing the caspase cascade. Gefitinib caused a rapid increase in the level of the proapoptotic BH3-only protein BIM (also called BCL2-like 11) through both transcriptional and post-translational mechanisms. Experiments with pharmacological inhibitors indicated that blockade of MEK–ERK1/2 (mitogen-activated protein kinase kinase–extracellular signal-regulated protein kinase 1/2) signaling, but not blockade of PI3K (phosphatidylinositol 3-kinase), JNK (c-Jun N-terminal kinase or mitogen-activated protein kinase 8), or AKT (protein kinase B), was critical for BIM activation. Using RNA interference, we demonstrated that BIM is essential for gefitinib-induced killing of NSCLC cells. Moreover, we found that gefitinib-induced apoptosis is enhanced by addition of the BH3 mimetic ABT-737.

**Conclusions:**

Inhibitors of the EGFR tyrosine kinase have proven useful in the therapy of certain cancers, in particular NSCLCs possessing activating mutations in the EGFR kinase domain, but the mechanisms of tumor cell killing are still unclear. In this paper, we demonstrate that activation of the proapoptotic BH3-only protein BIM is essential for tumor cell killing and that shutdown of the EGFR–MEK–ERK signaling cascade is critical for BIM activation. Moreover, we demonstrate that addition of a BH3 mimetic significantly enhances killing of NSCLC cells by the EGFR tyrosine kinase inhibitor gefitinib. It appears likely that this approach represents a paradigm shared by many, and perhaps all, oncogenic tyrosine kinases and suggests a powerful new strategy for cancer therapy.

## Abstract 2

Andreas Strasser and colleagues demonstrate that activation of the proapoptotic BH3-only protein BIM is essential for tumor cell killing and that shutdown of the EGFR−MEK−ERK signaling cascade is critical for BIM activation.

## Editors' Summary

**Background.:**

Normally, cell division (which produces new cells) and cell death are finely balanced to keep the human body in good working order. But sometimes cells acquire changes (mutations) in their genetic material that allow them to divide uncontrollably to form cancers—life-threatening, disorganized masses of cells. One protein with a critical role in cell division that is often mutated in tumors is the epidermal growth factor receptor (EGFR). In normal cells, protein messengers bind to EGFR and activate its tyrosine kinase. This enzyme then adds phosphate groups to tyrosine (an amino acid) in proteins that form part of signaling cascades (for example, the MEK–ERK signaling cascade) that tell the cell to divide. In cancers that have mutations in EGFR, signaling is overactive so the cancer cells divide much more than they should. Some non-small cell lung cancers (NSCLC, the commonest type of lung cancer), for example, have activating mutations within the EGFR tyrosine kinase. Treatment with EGFR tyrosine kinase inhibitors (TKIs) such as gefitinib and erlotinib induces the cells in these tumors to stop growing and die. This cell death causes tumor shrinkage (regression) and increases the life expectancy of patients with this type of NSCLC.

**Why Was This Study Done?:**

Unfortunately, treatment with TKIs rarely cures NSCLC, so it would be useful to find a way to augment the effect that TKIs have on cancer cells. To do this, the molecular mechanisms that cause cancer-cell death and tumor regression in response to these drugs need to be fully understood. In this study, the researchers have used a combination of biochemical and genetic approaches to investigate how gefitinib kills NSCLC cells with mutated EGFR.

**What Did the Researchers Do and Find?:**

The researchers first measured the sensitivity of NSCLC cell lines (tumor cells that grow indefinitely in dishes) to gefitinib-induced apoptosis. Gefitinib caused extensive apoptosis in two cell lines expressing mutant EGFR but not in one expressing normal EGFR. Next, they investigated the mechanism of gefitinib-induced apoptosis in the most sensitive cell line (H3255). Apoptosis is activated via two major pathways. Hallmarks of the “intrinsic” pathway include activation of a protein called BAX and cytochrome *c* release from subcellular compartments known as mitochondria. Gefitinib treatment induced both these events in H3255 cells. BAX (a proapoptotic member of the BCL-2 family of proteins) is activated when proapoptotic BH3-only BCL-2 proteins (for example, BIM; “BH3-only” describes the structure of these proteins) bind to antiapoptotic BCL2 proteins. Gefitinib treatment rapidly increased BIM activity in H3255 and HCC827 cells (but not in gefitinib-resistant cells) by increasing the production of BIM protein and the removal of phosphate groups from it, which increases BIM activity. Pharmacological blockade of the MEK–ERK signaling cascade, but not of other EGFR signaling cascades, also caused the accumulation of BIM. By contrast, blocking BIM expression using a technique called RNA interference reduced gefitinib-induced apoptosis. Finally, a combination of gefitinib and a BH3-mimicking compound called ABT-737 (which, like BIM, binds to antiapoptotic BCL-2 proteins) caused more apoptosis than gefitinib alone.

**What Do These Findings Mean?:**

These findings (and those reported by Gong et al. and Costa et al.) indicate that activation of the proapoptotic BH3-only protein BIM is essential for gefitinib-induced killing of NSCLC cells that carry EGFR tyrosine kinase mutations. They also show that inhibition of the EGFR–MEK–ERK signaling cascade by gefitinib is essential for BIM activation. Because these findings come from studies on NSCLC cell lines, they need confirming in freshly isolated tumor cells and in tumors growing in people. However, the demonstration that a compound that mimics BH3 action enhances gefitinib-induced killing of NSCLC cells suggests that combinations of TKIs and drugs that affect the intrinsic pathway of apoptosis activation might provide a powerful strategy for treating cancers in which tyrosine kinase mutations drive tumor growth.

**Additional Information.:**

Please access these Web sites via the online version of this summary at http://dx.doi.org/10.1371/journal.pmed.0040316.

- A perspective by Ingo Mellinghoff discusses this article and two related research articles
- Wikipedia pages on epidermal growth factor receptor, apoptosis, and BCL2 proteins (note that Wikipedia is a free online encyclopedia that anyone can edit; available in several languages)
- CancerQuest provides information on all aspects of cancer</ext-link> from Emory University (in several languages)
- US National Cancer Institute information for patients and professionals on lung cancer (in English and Spanish)
- Information for patients from Cancer Research UK on lung cancer including information on treatment with TKIs
- Information for patients from Cancerbackup on erlotinib and gefitinib

## Introduction

The epidermal growth factor receptor (EGFR) is a type I surface-bound receptor tyrosine kinase of the ErbB receptor family. Its activation by physiological ligands (e.g., EGF) causes EGFR homodimerization or heterodimerization of EGFR with other members of the ErbB family, resulting in activation of diverse signaling molecules such as extracellular signal-regulated protein kinase 1/2 (ERK1/2), protein kinase B (AKT), and signal transducer and activator of transcription proteins (STATs), which regulate cellular proliferation, survival, differentiation, and migration (reviewed in [1]). EGFR function is commonly dysregulated in a range of solid cancers (e.g., breast, lung, ovarian, bladder, brain, and colon) due to either gene amplification, mutations (resulting in a constitutively active EGFR), or abnormally increased ligand production (reviewed in [1]). Moreover, enforced expression of mutant EGFR in transgenic mice promoted development of lung carcinomas [2,3]. These observations prompted the development of EGFR inhibitory drugs for cancer therapy. The EGFR tyrosine kinase inhibitors gefitinib (Iressa, AstraZeneca) and erlotinib (Tarceva, Genentech) as well as the monoclonal antibody cetuximab (Erbitux, Merck), which blocks ligand binding, cause substantial regression of a small proportion of non-small cell lung cancers (NSCLCs), particularly those with *EGFR* mutations that give rise to hyperactive kinases [1,4–6]. Signaling from mutant but not wild-type (WT) EGFR was shown to activate anti-apoptotic pathways, and small interfering RNA-mediated down-regulation of mutant EGFR resulted in the death of these cells [7], but the mechanisms for tumor cell killing were not examined.

Mammals have two distinct but ultimately converging apoptosis signaling pathways [8], the extrinsic pathway, which is activated by “death receptors,” and the intrinsic (also called “mitochondrial” or “BCL-2-regulated”) pathway [9]. The BCL-2 family of proteins regulate the intrinsic apoptosis signaling pathway, and according to their structure and function they can be divided into three groups. The BAX- and BAK-like proteins, which share three regions of homology (BCL-2-homology [BH] domains), are proapoptotic and perturb the mitochondrial membrane potential when activated, resulting in release of cytochrome *c*, activation of the caspase cascade, and cellular destruction [10]. To prevent cell death, BAX and BAK are bound and inhibited by the antiapoptotic members of the BCL-2 family (BCL-2, BCL-x_L_, BCL-w, MCL-1, and A1), which share up to four BH regions [10]. The third subgroup, the BH3-only proteins (BAD, BID, BIK [also called BLK or NBK], HRK [also called DP5], BIM [also called BOD], NOXA, PUMA [also called BBC3], and BMF), share with each other and the remainder of the BCL-2 family only the 9- to 16-amino acid BH3 domain. The BH3-only proteins initiate apoptosis signaling by binding and antagonizing the prosurvival BCL-2 family members, thereby causing activation of BAX and BAK [11]. BH3-only proteins can be regulated by a range of transcriptional and post-translational mechanisms [12], and experiments with gene-targeted mice have shown that different members of this subgroup are required for the execution of different death stimuli. For example, PUMA and to a lesser extent NOXA are critical for DNA damage-induced apoptosis [13–15], whereas BIM is essential for hematopoietic cell homeostasis and cytokine deprivation-induced apoptosis [16].

Here, we studied the molecular mechanisms through which certain NSCLC tumor cell lines expressing mutant but not wild-type (WT) EGFR undergo apoptosis after treatment with the EGFR inhibitor gefitinib.

## Methods

### Cell Lines, Expression Vectors, and Cell Transfection

The NSCLC cell lines NCI-H358, NCI-441, NCI-H1650, and NCI-H1975 were all obtained from ATCC. NCI-H3255 cells were obtained from Drs. Bruce Johnson and Kreshnik Zejnullahu (Dana Faber, Boston). (NCI is left out from the nomenclature hereafter). HCC827 cells were a kind gift of Dr. Dan Costa (Department of Medicine, Harvard Medical School, Boston, MA). H358 and H441 cells express WT EGFR, whereas H1650 and HCC827 cells harbor an exon 19 mutation (DelE746A750) and H3255 cells possess a single amino acid substitution mutation (L858R) in the *EGFR* gene. H1975 cells harbor two mutations (L858R and T790M) in the *EGFR* gene. All cells were maintained in RPMI-1640 medium supplemented with 10% heat-inactivated fetal calf serum (FCS, JRH Biosciences, Lenexa, Kansas). The caspase inhibitor QVD-OPH (MP Biomedicals, Aurora, Ohio) was used at 25 μM and added to cells 30 min prior to treatment with gefitinib. Kinase inhibitors UO126, PD98059 (CST, Beverley, MA), SP6, LY294002, AKT inhibitor Akti1/2 (Merck), ABT-737, gefitinib (AstraZeneca), erlotinib, and cetuximab (gifts from Dr. Thomas Valerius, University of Schleswig-Holstein, Germany) were dissolved in DMSO and used as indicated. The anti-*BIM* short hairpin RNA construct, cloned into pSUPER with the neomycin-resistant gene, has been described previously [17]. Transfection with Fugene (Roche, Indianapolis, IN) was performed according to the manufacturer's instructions and vector-transfected clones were selected with 1 mg/ml Geneticin (Gibco BRL, Grand Island, NY). Cell lines were single cell-cloned by limiting dilution.

### Western Blotting

Protein samples were separated by SDS-PAGE and then electroblotted onto a PVDF membrane (Hybond P, Amersham Biosciences). Antibodies against BCL-w (clone 13F9; Alexis), BCL-x_L_ (BD/Pharmingen), BIM (clone 3C5, Alexis; or polyclonal antibodies from Stressgen), BAD (Stressgen), phospho-BAD (phosphorylated at Ser112), phospho-BAD (Ser136), phospho-ERK1/2 (Thr202/Tyr204), total ERK1/2, phospho-AKT (193H12, Ser473), total AKT, phospho-EGFR (Tyr1068), total EGFR (all from CST), BAX (Upstate), human BMF (polyclonal antibody from Alexis), HSP70 (N6; gift from Dr. R Anderson, Peter MacCallum Cancer Institute, Melbourne, Australia), MCL-1 (Dako), PARP (Alexis), PUMA (NT, Pro-Sci), and β-actin (Sigma) were used as indicated by the manufacturers. Detection was performed with horseradish peroxidase-conjugated secondary antibodies (specific to rat, mouse, hamster, or rabbit IgG) and enhanced chemiluminescence (Amersham Biosciences).

### Two-Dimensional Gel Electrophoresis

Two-dimensional protein gel electrophoresis was performed using the IPGphor isoelectric focusing system (Amersham Biosciences). BIM was isolated from cell lysates by immunoaffinity chromatography using mAb 3C5 (Alexis). Samples were then loaded onto IPG gels, rehydrated at 20 °C for 12 h, and subjected to isoelectric focusing for at least 22 h. After equilibration with SDS-PAGE buffer for 15 min at room temperature, the IPG gel was subjected to SDS-PAGE. Transfer, immunoblotting and visualization were performed as described above.

### Reverse Transcriptase Polymerase Chain Reaction

Total RNA was extracted using the Micro-to-Midi Total RNA Extraction Kit (Invitrogen), and total RNA subjected to reverse transcription. For semiquantitative analysis, cDNA was subjected to PCR, using primers for *BIM*, with samples removed after a given number of cycles. Primers for *BIM* were used as previously described [18]. β-actin was used as a control for the quality and abundance of RNA. For quantitative PCR analysis of *BIM* and *PUMA* expression, TaqMan probes were used in conjunction with an ABI-PRISM 7900 thermal cycler (Applied Biosystems) according to the manufacturer's instructions. β2 microglobulin was used as a control for the quality and abundance of RNA. Fold induction of *BIM* and *PUMA* was calculated by comparing Ct values of treated and untreated samples after first correcting for RNA abundance.

### Cell Death Assays

Cell death was assessed following release of the cells from the culture dish through trypsinization. Cell death was assessed by flow cytometric analysis in a FACScan (Becton Dickinson), either by staining with propidium iodide (PI) plus Annexin V-FITC or by assessing the extent of DNA fragmentation as detailed previously [19]**.** The latter technique was also performed to assess changes in cell cycle distribution.

### Assays for BAX Activation

Activation status of BAX was assessed using the activation-specific mAb for BAX, followed by flow cytometric analysis as detailed previously [20]. Alternatively, BAX activation was assessed by following its redistribution from the cytosolic to the membrane fraction by subcellular fractionation using digitonin lysis followed by SDS-PAGE and Western blotting, as detailed previously [20]. Membranes were also probed to determine whether cytochrome *c* had been released from the mitochondria (redistribution from the membrane to the cytosolic fraction) and for BAK (membrane-resident) as well as HSP70 (cytosol-resident) as controls for the quality of subcellular fractionation and protein loading.

## Results

### Varying Sensitivities of NSCLC Cell Lines to Gefitinib-Induced Apoptosis

**Figure 1:**
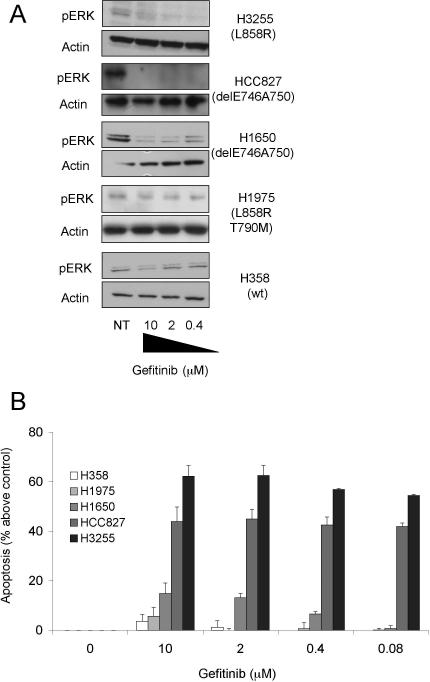
Effect of Gefitinib on NSCLC Cells Expressing WT or Mutant *EGFR* NSCLC cells expressing WT (H358) or mutant (HCC827, H1975, H1650, H3255) *EGFR* were treated with varying concentrations of gefitinib for 24 h (A) or 72 h (B). Cells were then either assessed for the phosphorylation status of ERK (A) or cell death (B). Western blotting (A) was performed to determine the phosphorylation status of ERK1/2 before and after treatment with gefitinib. An actin loading control is also shown. Cell death was assessed by Annexin V-FITC plus PI staining (B). Results represent mean ± standard error of the mean (SEM) of at least three experiments. NT, no treatment.

The NSCLC cell lines H358 (WT EGFR), H1650, HCC827 (Del E746A750 deletions) and H3255 (L858R mutation) [5,7,21,22] were chosen for initial studies on the effects of gefitinib. Gefitinib potently inhibited the activation of ERK1/2 in all three cell lines expressing mutant EGFR, as judged by its dephosphorylation, whereas the H358 cells expressing WT EGFR were unaffected, as previously reported (Figure 1A) [5,7,21,22]. Extensive apoptosis was observed only in H3255 and HCC827 cells (Figure 1B). H1650 cells displayed only a low level of apoptosis and H358 cells were refractory. In addition, we also examined H1975 cells, which were originally reported as gefitinib sensitive [7]. However, we found that gefitinib treatment of these cells did not result in dephosphorylation of ERK1/2 (Figure 1A) or substantial apoptosis (Figure 1B), presumably due to the presence of the additional (T790M) mutation, known to reduce the kinase activity of EGFR, rendering it effectively wild type [23]. Next, we assessed the mechanism of cell death in the highly sensitive H3255 cells.

### Mechanisms of Gefitinib-Induced Apoptosis

Apoptosis after treatment of H3255 cells with gefitinib featured PARP cleavage and phosphatidylserine exposure (as detected by staining with FITC-coupled Annexin V), was caspase dependent and involved activation of BAX and mitochondrial release of cytochrome *c* (Figure S1). BAX activation was revealed both by flow cytometric analysis using antibodies that recognize activated BAX and by subcellular fractionation, which showed that a substantial proportion of BAX redistributed from the cytosol to the mitochondrial fraction after treatment with gefitinib (Figure S1D and S1E).

**Figure 2:**
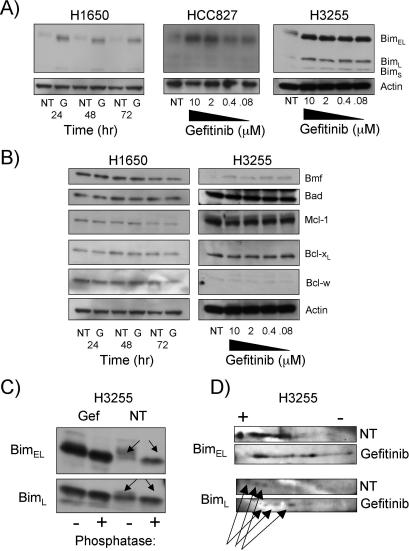
Induction and Dephosphorylation of BIM after Gefitinib Treatment in NSCLC Cells Expressing Mutant EGFR (A) In the blot on the left, H1650 cells were left untreated (NT) or treated with 1 μM gefitinib (G) for 24, 48, or 72 h and the cells harvested, lysed, and assessed by Western blotting for the expression of BIM. HCC827 or H3255 cells (center and right, respectively) were treated for 16 h with a range of concentrations of gefitinib (10–0.08 μM) and the expression of BIM analyzed as above. Actin is shown as a loading control. (B) H1650 or H3255 cells were treated as in (A) and then BH3-only proteins, prosurvival BCL-2 family members, or β-actin (loading control) assessed by Western blotting. (C) H3255 cells were left untreated (NT) or treated for 16 h with 1 μM gefitinib (Gef) and the cells harvested and lysed. Portions of the samples were then treated with λ-phosphatase (+) or left untreated (−) and then assessed by Western blotting for BIM (labeled Bim_EL_ and Bim_L_). (D) H3255 cells were left untreated or treated for 16 h with 1 μM gefitinib and the cells harvested and lysed. BIM (labeled Bim_EL_ and Bim_L_) was isolated by immunoaffinity chromatography and subjected to two-dimensional gel SDS-PAGE followed by Western blotting for BIM.

BAX and BAK are activated when prosurvival BCL-2 family members are antagonized by the proapoptotic BH3-only proteins [11]. In all three mutant EGFR-expressing cell lines, gefitinib caused a significant induction of BIM in a time- and dose-dependent manner (Figures 2A, S2, and S3). BIM has a number of isoforms, the major ones designated BIM_EL_ (extra long), BIM_L_ (long), and BIM_S_ (short), which are generated through alternative splicing and therefore differ in molecular weight [24]. We routinely observed BIM_EL_ and BIM_L_ isoforms in our gefitinib-treated cells, with BIM_EL_ the most highly expressed (Figure 2A). In accordance with the differential sensitivity of the cell lines to gefitinib-induced apoptosis, BIM induction was more prominent in H3255 cells compared to the HCC827 and H1650 cells (Figure 2A). The expression of other BH3-only proteins (BMF, BAD, and PUMA) and the prosurvival BCL-2 family members BCL-w, BCL-x_L_, and MCL-1 did not change significantly in either the H3255 or H1650 cells after treatment with gefitinib (Figure 2B). Notably, BIM was not strongly induced in two NSCLC cell lines expressing WT EGFR or in the H1975 cells expressing the L858R and T790M mutant *EGFR* (Figure S4). Therefore, the extent of BIM induction after gefitinib treatment correlated directly with the extent of apoptosis induced in the NSCLC cells.

### BIM Is Essential for Gefitinib-Induced Apoptosis of NSCLC Cells

**Figure 3:**
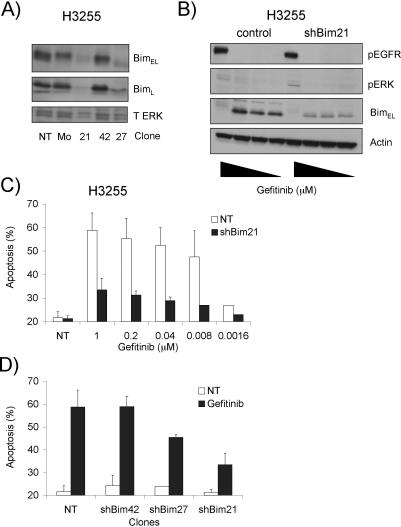
*BIM* Knockdown by RNAi Protects H3255 Cells against Gefitinib-Induced Killing (A) Western blot analysis documents the level of *BIM* expression in parental and *BIM* RNAi knockdown subclones of H3255 cells. Probing with an antibody to total ERK (T ERK) was used as a loading control. (B and C) Parental and *BIM* RNAi knockdown subclone #21 of H3255 cells were left untreated (NT) or treated for 18 h with gefitinib (1–0.04 μM) prior to cell lysis and Western blotting (B) or assessment of cell death (C). Blots (B) were probed with antibodies specific to phosphorylated EGFR (pEGFR), phosphorylated ERK (pERK), BIM or β-actin (loading control). Cell viability (C) was determined by staining with Annexin V-FITC plus PI, followed by flow cytometric analysis. (D) Parental and *BIM* RNAi knockdown subclones (#21, #27, and #42) of H3255 cells were treated for 24 h with DMSO (vehicle control; NT) or 1 μM gefitinib and cell death assessed as above. Data represent means ± SEM of three experiments.

To examine the role of BIM in gefitinib-induced cell killing, we generated multiple subclones of H3255 cells stably expressing a *BIM* RNA interference (RNAi) construct. In these transfectants BIM expression was reduced, even after treatment with gefitinib (Figure 3A and 3B). *BIM* knockdown protected H3255 cells potently against gefitinib over a range of concentrations, and the level of protection correlated with the extent of BIM reduction in these subclones (Figure 3C and 3D). The *BIM* RNAi transfectants still responded to gefitinib, as judged by its ability to elicit dephosphorylation of EGFR as well as its downstream targets ERK1/2 and to induce G1 cell cycle arrest (Figure 3B and unpublished data).

### Gefitinib Causes Increased *BIM* Transcription as well as Post-translational Modifications in BIM

The proapoptotic activity of BIM can be regulated by a range of transcriptional and post-translational mechanisms [12]. Semiquantitative reverse transcriptase PCR and quantitative PCR analyses demonstrated that gefitinib induced a substantial (∼3-fold) increase in *BIM* mRNA in H3255 cells and to a lesser extent in H1650 cells (Figures S5A and S5B). The induction of *BIM* was specific, as expression of other BH3-only genes was not elevated and the levels of *PUMA* mRNA actually fell after gefitinib treatment (Figure S5C).

The electrophoretic mobility of BIM_EL_ changed rapidly (within 15–60 min) after gefitinib treatment of H3255 cells, coinciding with loss of ERK1/2 activity (Figure S6A). BIM_EL_ has multiple phosphorylation sites and its proapoptotic activity can be down-regulated through ERK-mediated phosphorylation, which targets it for ubiquitination and proteasomal degradation [12,25,26]. Treatment of cell lysates with λ phosphatase showed that BIM_EL_ and BIM_L_ are phosphorylated in healthy but not in gefitinib-treated cells (Figure 2C). Two-dimensional gel electrophoresis and Western blotting demonstrated that BIM_EL_ and BIM_L_ immunoprecipitated from gefitinib-treated H3255 cells had less negative charge than BIM from untreated cells, confirming the accumulation of dephosphorylated forms of BIM (Figure 2D). The levels of phosphorylated BAD (Ser136 but not Ser112) dropped after gefitinib treatment (Figure S6B), likely as a consequence of shutdown of the AKT and/or ERK1/2 pathways, which are known to promote phosphorylation of BAD and thereby inhibit its proapoptotic activity [27]. However, significant BAD dephosphorylation occurred only when apoptosis was already underway, perhaps indicating that it is a consequence rather than an initiator of cell death. Unfortunately, our attempts to decrease *BAD* expression in the H3225 cells by RNAi have so far failed, so the importance of BAD in gefitinib-induced death remains to be determined.

### Signal Transduction Pathways Leading to BIM Activation That Are Affected by Gefitinib

**Figure 4:**
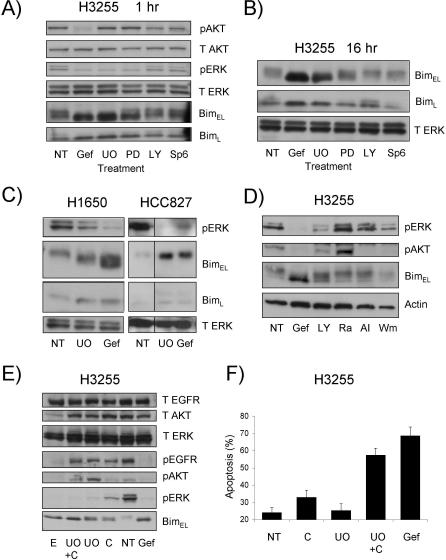
Role of Shutdown of the MEK, JNK, PI3K, and AKT Signaling Pathways in BIM Dephosphorylation and Accumulation (A and B) H3255 cells were treated with UO126 (UO, 20 μM), PD98059 (PD, 20 μM), LY294002 (LY, 25 μM), Sp6 (20 μM), or gefitinib (Gef, 1 μM) for 1 h (A) or 16 h (B) and then harvested, lysed, and assessed by Western blotting. Blots were probed for phosphorylated AKT (pAKT), total AKT (T AKT), phosphorylated ERK (pERK), total ERK (T ERK) and BIM. (C) H1650 and HCC827 cells were treated for 24 h with either UO126 (20 μM) or gefitinib (1 μM) and then harvested, lysed, and assessed by Western blotting for the levels of BIM, phosphorylated ERK (pERK), and total ERK (T ERK). (D) H3255 cells were treated for 16 h with gefitinib (Gef, 1 μM), LY294002 (LY, 25 μM), Wortmannin (Wm, 1 μM), AKT inhibitor (AI, 20 μM), or rapamycin (Ra, 100 ng/ml) and then harvested, lysed, and assessed by Western blotting as in (A). (E) H3255 cells were treated for 16 h with various combinations of cetuximab (C, 10 μg/ml), UO126 (20 μM), erlotinib (E, 1 μM), or gefitinib (Gef, 1 μM) and then harvested, lysed, and assessed by Western blotting as in (A). (F) H3255 cells were treated for 48 h with various combinations of cetuximab (C, 10 μg/ml), UO126 (20 μM), or gefitinib (Gef, 1 μM) and then cell death assessed as previously. Data represent means ± SEM of at least three experiments.

To identify the critical pathways responsible for *BIM* transcriptional induction and BIM dephosphorylation after gefitinib treatment, we employed a range of specific inhibitors to block MEK (UO126 or PD98059), PI3K (LY294002), or JNK (SP6). These studies revealed that MEK inhibition alone was sufficient to induce rapid (within 1 h) dephosphorylation of BIM_EL_ and BIM_L_, whereas PI3K and JNK inhibitors had no effect (Figure 4A). Although the extent of BIM induction after 16 h of treatment correlated with the potency of MEK inhibition (UO126 is a more potent inhibitor than PD98059 [28]), it was not able to cause BIM up-regulation to the extent seen with gefitinib (Figure 4B). Similar results were also observed in H1650 and HCC827 cells (Figure 4C) in which MEK inhibition caused an increase in the expression and dephosphorylation of BIM_EL_ but to a lesser extent than that induced with gefitinib. Therefore, although MEK–ERK1/2 inhibition appears important for BIM dephosphorylation and accumulation, other signaling pathways are involved to achieve maximal BIM induction. The obvious candidate would be the PI3K–AKT pathway. Surprisingly, the well-established PI3K inhibitor LY294002 caused only incomplete AKT dephosphorylation in the H3255 cells, in contrast to gefitinib, which achieved almost complete AKT dephosphorylation (Figure 4A). Therefore, to explore the importance of the PI3K/AKT pathway further, we employed a number of additional inhibitors of PI3K (Wortmannin), mTor (rapamycin), or AKT (Akti1/2) [29]. Although Wortmannin and the AKT inhibitor induced complete AKT dephosphorylation, they did not result in BIM accumulation (Figure 4D). This indicates that effects of gefitinib on the PI3K–AKT pathway may not be required for full BIM induction in NSCLC cells, but it remains possible that AKT triggers other antiapoptotic pathways (e.g., BAD inactivation) in these cells. This suggestion is supported by the observation that although BIM levels did not rise substantially upon treatment with either PI3K or AKT inhibitors, both induced apoptosis (unpublished data).

### EGFR Inhibitors Other than Gefitinib also Cause BIM Induction in NSCLC Cell Lines

Since gefitinib caused activation of BIM in NSCLC cells harboring *EGFR* mutations, we investigated whether other EGFR inhibitors had similar effects. Addition of cetuximab (a monoclonal antibody inhibitor of EGFR) resulted in the induction of BIM, although to a lower extent compared to treatment with gefitinib. This correlated with lower levels of inactivation (dephosphorylation) of EGFR and ERK1/2 (Figure 4E) and also less cell killing (Figure 4F), in line with recent reports [30]. In contrast, a second EGFR tyrosine kinase inhibitor, erlotinib, achieved levels of inactivation of EGFR, AKT, and ERK similar to gefitinib and resulted in comparable levels of BIM induction, BIM dephosphorylation (Figure 4E), and cell death (unpublished data). Combining the MEK inhibitor UO126 with cetuximab resulted in increased levels of BIM (Figure 4E) and cell death (Figure 4F) compared to treatment with cetuximab alone, illustrating the importance of inhibiting the ERK1/2 signaling pathway for efficient BIM accumulation. However, it should be noted that gefitinib reproducibly induced more cell death than did the combination of cetuximab and UO126 (Figure 4F), indicating that other MEK-ERK–independent signaling pathways also contribute to cell death after gefitinib treatment.

### EGFR Inhibitors and the BH3-Mimetic ABT-737 Synergize in the Killing of NSCLC Cell Lines

**Figure 5:**
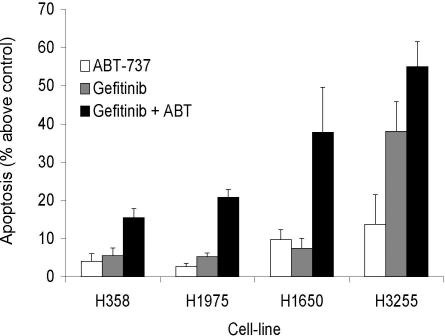
Synergy between Gefitinib and ABT-737 in Killing of NSCLC Cells Expressing Activating Mutations of *EGFR* H358, H1650, H1975, and H3255 cells were treated with gefitinib (H358, H1975, and H1650, 10 μM gefitinib; H3255, 1 μM gefitinib) in the presence or absence of ABT-737 (1 μM) for 32 h (H3255 cells) or 48 h (H358, H1650, H1975 cells). Cells were then harvested and survival measured as in Figure 1B. Data represent means ± SEM of three experiments indicating percentage of cell death compared to untreated cells.

On their own, EGFR inhibitors such as gefitinib or erlotinib are unlikely to provide cures in the majority of NSCLC patients, even those harboring mutant *EGFR*. Therefore, we explored how to augment the effects of these drugs. BH3 mimetics such as ABT-737 bind with high affinity to BCL-2, BCL-x_L_, and BCL-w, and kill certain tumor cells when used alone or in combination with chemotherapeutic drugs or γ-irradiation [31]. We found that ABT-737 substantially enhanced gefitinib-induced apoptosis in H3255 cells and even in the relatively insensitive H1650 cells (Figure 5). Furthermore, ABT-737 modestly enhanced the gefitinib-induced apoptosis of the H1975 cells. These results therefore demonstrate that EGFR inhibitors and the BH3 mimetic ABT-737 synergize in the killing of NSCLC cell lines.

## Discussion

In this paper, we demonstrate that activation of the proapoptotic BH3-only protein BIM is essential for tumor cell killing and that shutdown of the EGFR–MEK–ERK signaling cascade is critical for BIM activation. Moreover, we demonstrate that addition of a BH3 mimetic significantly enhances killing of NSCLC cells by the EGFR tyrosine kinase inhibitor gefitinib.

Recent data have highlighted the fact that inhibitors directed to critical receptors, kinases, and enzymes that are dysregulated during tumorigenesis present unique and powerful targets for cancer therapy. A prime example is the BCR-ABL fusion product, which results from the reciprocal t(9;22)(q34;q22) chromosomal translocation (Philadelphia [Ph^1^] chromosome), essential for development and sustained growth of chronic myelogenous leukemia and Ph^1^-positive (Ph^1^+) acute lymphoblastic leukemias [32]. Inhibition of BCR-ABL using the specific kinase inhibitor imatinib (Gleevec) results in cell death and tumor regression [33]. Recently, we showed that the cell death pathway evoked is critically dependent on BIM with supporting roles for BAD and BMF [34].

Here, we show that inhibition of the mutant EGFR found in certain NSCLC cells triggers cell death through a similar mechanism. We also show that three NSCLC cell lines expressing activating mutants of *EGFR*, but not cell lines expressing wild-type *EGFR* or mutant *EGFR* with wild-type signaling potential (H1975), are sensitive to apoptosis induced by the EGFR inhibitor gefitinib. Of the three cell lines with the hyperactive EGFR, H1650 cells were much less sensitive than the H3255 and HCC827 cells, and apparently much less sensitive to EGFR inhibition than previously reported, where 90% cell death was induced after small interfering RNA knockdown of the mutant *EGFR* [7]. Two independent vials of H1650 (obtained from ATCC) displayed the same sensitivity to gefitinib. For this reason we can only assume that genetic drift has occurred from the original cells reported in the earlier work. Notably, similar gefitinib resistance of this cell line has been reported independently (Costa and Kobayashi, personal communication). Potentially, protection from gefitinib-induced apoptosis in H1650 cells relates to its higher level of pAKT and/or pERK1/2 (unpublished data), since an inability of gefitinib to block AKT and/or ERK1/2 activation has previously been linked to apoptosis resistance in NSCLC cells [21,35].

Death of the sensitive H3255 cells was associated with the subcellular redistribution and activation of BAX, and was caspase dependent as judged by the clear PARP cleavage and inhibition of apoptosis by the caspase inhibitor QVD-OPH. Activation of BAX was more readily apparent in the presence of QVD-OPH, presumably due to the inhibition of cell destruction and consequent BAX degradation. This result is consistent with the view that BAX is activated through a caspase-independent pathway and is situated upstream of caspase activation in the cell death pathway.

Western blot analysis revealed that gefitinib consistently induced BIM in all three NSCLC lines expressing mutant *EGFR*. The level of BIM induction was higher in the H3255 and HCC827 cells, in accordance with their greater sensitivity to apoptosis than the H1650 cells. BIM induction preceded apoptosis, and RNAi-mediated *BIM* knockdown protected H3255 cells potently from gefitinib with the level of protection correlated with the extent of BIM reduction in various subclones. These results demonstrate that BIM is essential for the initiation of gefitinib-induced apoptosis in these cells. Similar dependence on BIM for gefitinib-induced apoptosis of HCC827 cells has been observed independently (Costa and Kobayashi, personal communication).

The induction of BIM was a consequence of both transcriptional induction and post-translational modification. Post-translational regulation of BIM after gefitinib treatment involved rapid dephosphorylation and was a result of ERK1/2 inhibition, downstream of MEK shutdown. ERK1/2 kinases are known to phosphorylate BIM and regulate its turnover in cells by targeting it for ubiquitination and subsequent proteasomal degradation [26,36,37]. Therefore, inhibition of ERK1/2 provides a mechanism through which EGFR blockade by gefitinib elicits BIM accumulation. Although gefitinib-induced dephosphorylation of BIM could be mimicked by treatment with MEK inhibitors, this did not cause BIM up-regulation (or apoptosis) to the extent seen with gefitinib. Therefore, although MEK–ERK1/2 inhibition appears critical for BIM dephosphorylation and accumulation, other signaling pathways must contribute to achieve maximal BIM induction and apoptosis. The identity of these pathways remains to be determined.

Imatinib-induced transcriptional up-regulation of BIM in BCR-ABL transformed cells was previously reported to be mediated by FOXO3A [38] and linked to the shutdown of PI3K–AKT signaling [39]. Because mutant EGFR potently stimulates the AKT pathway [7], we anticipated that a similar mechanism of BIM induction would be activated after gefitinib treatment of NSCLC cells expressing hyperactive mutant EGFR. However, although AKT phosphorylation was abrogated as a component of the response to gefitinib treatment, our data using pathway-specific inhibitors indicate that PI3K–AKT may not be involved. As such, neither the PI3K inhibitors LY294002 and Wortmannin nor a specific AKT inhibitor had any effect on BIM induction in NSCLC cells expressing mutant *EGFR*. This finding indicates that the PI3K–AKT–FOXO3 pathway is not critical for BIM induction after gefitinib treatment in these cells. However, this finding does not preclude the possibility that PI3K–AKT inactivation and consequent FOXO3A activation might play a role in the apoptosis observed after gefitinib treatment, as these inhibitors all triggered apoptosis in NSCLC cells (unpublished data). The downstream effectors of this apoptosis are unknown but are likely to include the BH3-only proteins BAD and PUMA, known targets of AKT [27] and FOXO3A [40], respectively. With regard to LY294002, it is noteworthy that this PI3K inhibitor did not substantially reduce AKT phosphorylation in these NSCLC cells, but still evoked apoptosis. Although surprising, this was a reproducible finding (with two different sources of the inhibitor), which indicates the presence of a signaling pathway regulated by PI3K but independent of AKT (and BIM). This pathway may involve the above-mentioned FOXO3A-mediated induction of the BH3-only protein PUMA [40].

A monoclonal antibody inhibitor of EGFR, cetuximab, induced BIM in H3255 cells, although to a lesser extent and with less dephosphorylation than that seen after treatment with either gefitinib or erlotinib. This effect correlated with a lower level of ERK1/2 dephosphorylation and less cell killing elicited by cetuximab than that achieved by gefitinib (Figure 4F). In agreement with this observation, cetuximab was previously shown to be less potent than gefitinib at inducing apoptosis in other *EGFR*-mutant NSCLC cells [30]. Accordingly, the combination of a MEK inhibitor with cetuximab resulted in increased BIM induction, comparable to that achieved by treatment with gefitinib, supporting the observation that MEK–ERK signaling is critical for BIM up-regulation in these cells. This combination of cetuximab plus MEK inhibitor also substantially enhanced apoptosis of the H3255 cells compared to either agent alone, but not to the level induced by gefitinib. This result supports the suggestion that other MEK–ERK-independent signaling pathways (likely those regulated by PI3K–AKT discussed above) also contribute to apoptosis induced by gefitinib in NSCLC harboring activating mutations of *EGFR*. More generally, these data reveal important differences between the signaling pathways triggered by EGFR kinase inhibitors and those induced by antagonistic monoclonal antibodies, and present a rational means for improving clinical responses to cetuximab - by combining it with MEK inhibitors.

Although initially promising it is now clear that EGFR inhibitors such as gefitinib or erlotinib are unlikely to provide cures in the majority of patients with NSCLC, even in those with cancers expressing mutant *EGFR*. However, understanding how these drugs work will provide critical information to help design strategies to augment their efficacy. Here we have shown that BIM up-regulation is essential for the apoptosis elicited by EGFR inhibitors in NSCLC cells harboring *EGFR* activating mutations, and detailed a number of the important mechanisms responsible. The next step will be to find strategies to augment the effects of the EGFR-inhibitory drugs. For example, blockade of signaling molecules within the Ras/MEK or PI3K/mTOR pathways might augment the effects of EGFR therapeutics, similar to their effects on chronic myelogenous leukemia cells when used in conjunction with the BCR-ABL inhibitor imatinib [41]. However, it should be noted that concurrent treatment of H3255, HCC827, or H1650 cells with gefitinib and a MEK inhibitor did not result in substantially enhanced apoptosis (unpublished data), presumably because gefitinib already efficiently inactivates ERK1/2 in these cells. Therefore, drugs that target the PI3K/mTOR pathways may be more successful.

Another therapeutic option is the use of BH3 mimetics, such as ABT-737 [31]. Here we showed that ABT-737 substantially enhanced gefitinib-induced apoptosis in all of the NSCLC cells tested, albeit most prominently in those harboring the EGFR activating mutations. This activity is reminiscent of the ability of ABT-737 to increase imatinib-induced apoptosis of BCR-ABL transformed cells [34]. We are currently assessing the efficacy of these combinations in suitable in vivo models in an attempt to inform subsequent clinical trials.

In conclusion, our results demonstrate that BIM is essential for gefitinib-induced killing of NSCLC cells expressing mutant *EGFR*. Shutdown of the MEK–ERK1/2 pathway appears critical for BIM up-regulation, but other signaling pathways may also contribute. Finally, we have shown that combining gefitinib with BH3 mimetics, such as ABT-737, may be a potent strategy for enhancing the currently suboptimal clinical responses seen in NSCLC.

## Supporting Information

Figure S1 Mechanism of Apoptosis Induced in H3255 Cells after Gefitinib Treatment (A) H3255 NSCLC cells were left untreated (NT) or treated with 1 μM gefitinib (Gef) or for 18 h. Cells were then harvested, lysed and Western blotted for PARP.
(B and C) H3255 cells were left untreated or incubated for 30 min with the caspase inhibitor QVD-OPH (25 μM) prior to the addition of gefitinib, and cell samples were assessed for cell death at 16 h or 24 h by Annexin V-FITC plus PI staining and flow cytometric analysis. Flow cytometry data from a representative experiment (B); mean ± standard deviation of three independent experiments (C).
(D and E) H3255 cells were left untreated (NT) or incubated for 30 min with QVD-OPH prior to the addition of gefitinib (Gef, 1μM) and cell samples assessed for BAX activation by flow cytometry at 16 or 24 h (D) or by subcellular localization (E) at 18 h assessing membrane (M) and cytosolic (C) compartments. Each fraction was assessed by Western blotting for cytochrome *c*, BAX, BAK, and HSP70 (the latter as a loading control).
(569 KB JPG)

Figure S2 BIM but not PUMA, BAX, or BAK Is Induced after Gefitinib Treatment in H3225 Cells and Is Coincident with PARP Cleavage H3255 cells were treated with 1 μM gefitinib for 5–15 h and the cells harvested, lysed and assessed by Western blotting for expression of PARP, PUMA, BIM, BAK, BAX, and actin (loading control).
(156 KB JPG)

Figure S3 Kinetics of BIM Induction after Gefitinib Treatment in HCC827 Cells Bim is induced after gefitinib treatment in HCC827 cells and is coincident with ERK dephosphorylation. HCC827 cells were treated with 1 μM gefitinib for 2–28 h and the cells harvested, lysed and assessed by Western blotting for expression of BIM, pERK, and actin (loading control).
(119 KB JPG)

Figure S4 Effect of Gefitinib on NSCLC Cells H358 and H441 cells expressing WT EGFR or H1975 cells expressing L858R and T790M mutant EGFR were left untreated (NT) or treated for 24 h with gefitinib (10, 2, 0.4, 0.08 μM) or DMSO (D). The cells were then assessed by Western blotting for the phosphorylation status of ERK1/2 and the level of BIM and actin (loading control).
(234 KB JPG)

Figure S5 *BIM* but Not *PUMA* mRNA Is Induced after Gefitinib Treatment in NSCLC Cells Expressing Mutant *EGFR*
NSCLC cells were treated with 1 μM gefitinib for 2, 6, or 24 h (H3255) or 24 h only (H1650). The cells were then harvested, total RNA isolated and converted to cDNA. Semiquantitative PCR (A) or quantitative PCR (B and C) analysis was then performed to determine the levels of BIM or PUMA. Bars represent the mean ± standard deviation of three independent experiments.
(410 KB JPG)

Figure S6 Inhibition of EGFR Results in the Up-regulation and Dephosphorlyation of BIM but Not BAD in H3225 Cells H3255 cells were left untreated (NT) or were treated with inhibitors of EGFR (1 μM gefitinib) for times ranging from 1 min to 16 h. The cells were then harvested, lysed and assessed by Western blotting for phosphorylated ERK1/2 (pERK1/2), BIM, phosphorylated BAD136, phosphorylated BAD112, total BAD, PARP, BCL-x_L_, or actin (loading control).
(A) The rapid kinetics of pERK loss and coincident decrease in BIM mobility on SDS-PAGE, indicative of dephosphorylation.
(B) The phosphorylation status of BAD and induction of BIM_EL_ and BIM_L_ over a 16 h time course. These data show that BAD136 but not BAD112 is dephosphorylated after 16 h treatment with gefitinib, coincident with PARP cleavage.
(379 KB JPG)

## Abbreviations

EGFR: epidermal growth factor receptor
ERK: extracellular signal-regulated protein kinase
MEK: mitogen-activated protein kinase kinase
NSCLC: non-small cell lung cancer
SEM: standard error of the mean
RNAi: RNA interference
WT: wild type
