# Supplementary figures and images for: Gefitinib-Induced Killing of NSCLC Cell Lines Expressing Mutant EGFR Requires BIM and Can Be Enhanced by BH3 Mimetics
Source: PLoS Med. 2007 Oct 30;4(10):e316. doi: 10.1371/journal.pmed.0040316 (PMC2043013; doi:10.1371/journal.pmed.0040316)

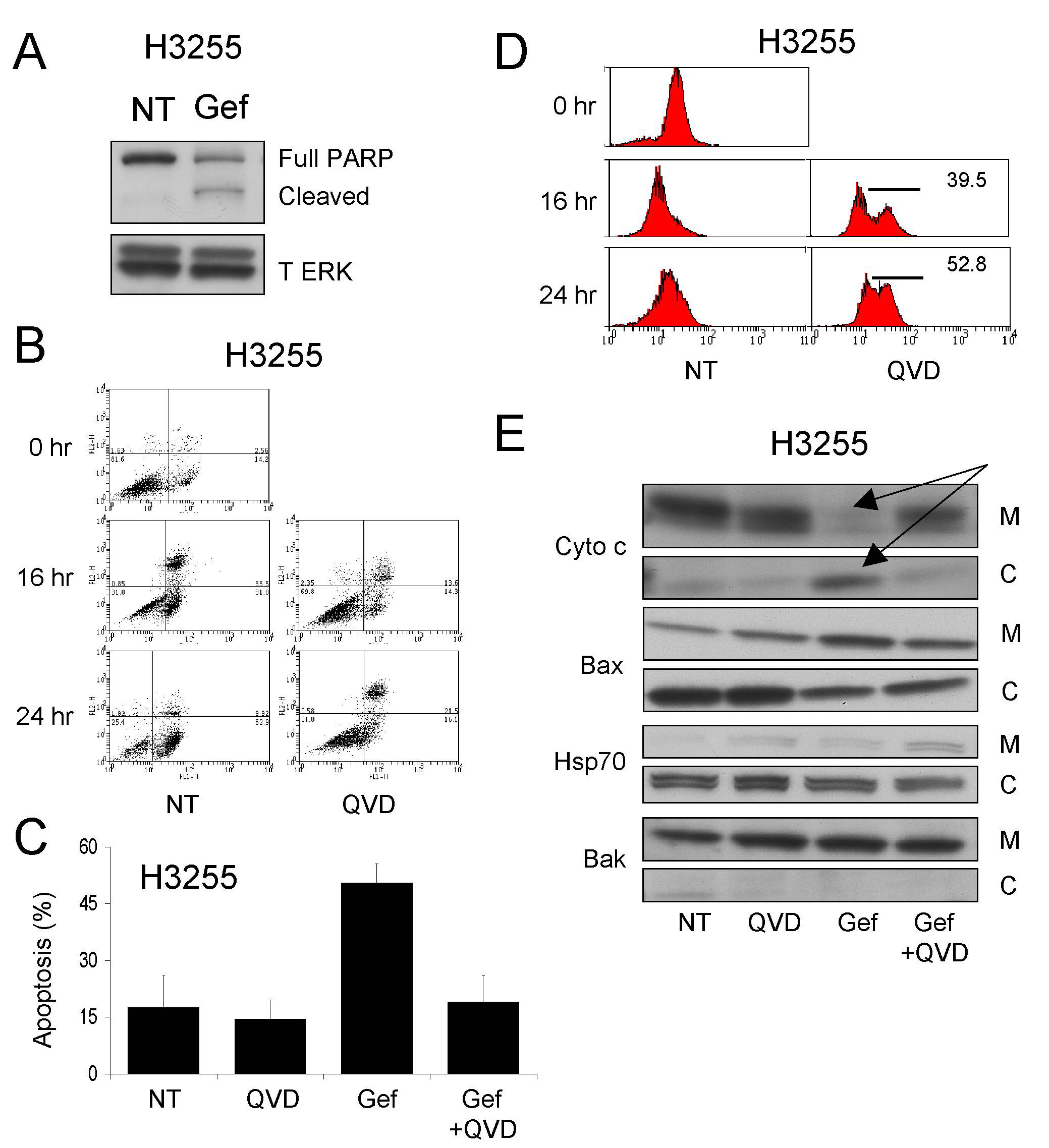

Supplement: Figure S1 — (A) H3255 NSCLC cells were left untreated (NT) or treated with 1 μM gefitinib (Gef) or for 18 h. Cells were then harvested, lysed and Western blotted for PARP. (B and C) H3255 cells were left untreated or incubated for 30 min with the caspase inhibitor QVD-OPH (25 μM) prior to the addition of gefitinib, and cell samples were assessed for cell death at 16 h or 24 h by Annexin V-FITC plus PI staining and flow cytometric analysis. Flow cytometry data from a representative experiment (B); mean ± standard deviation of three independent experiments (C). (D and E) H3255 cells were left untreated (NT) or incubated for 30 min with QVD-OPH prior to the addition of gefitinib (Gef, 1μM) and cell samples assessed for BAX activation by flow cytometry at 16 or 24 h (D) or by subcellular localization (E) at 18 h assessing membrane (M) and cytosolic (C) compartments. Each fraction was assessed by Western blotting for cytochrome c, BAX, BAK, and HSP70 (the latter as a loading control). (569 KB JPG) [file pmed.0040316.sg001.jpg]

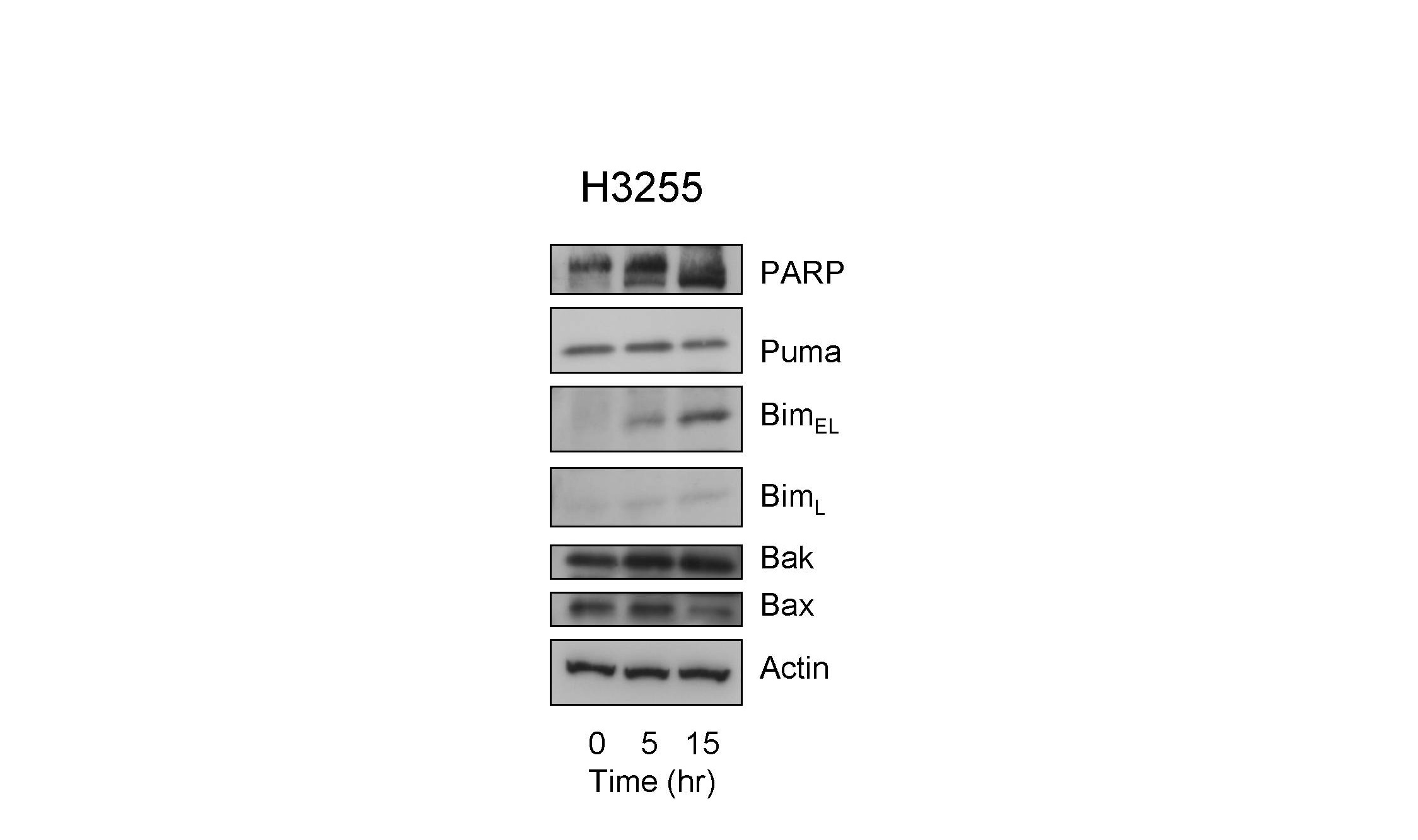

Supplement: Figure S2 — H3255 cells were treated with 1 μM gefitinib for 5–15 h and the cells harvested, lysed and assessed by Western blotting for expression of PARP, PUMA, BIM, BAK, BAX, and actin (loading control). (156 KB JPG) [file pmed.0040316.sg002.jpg]

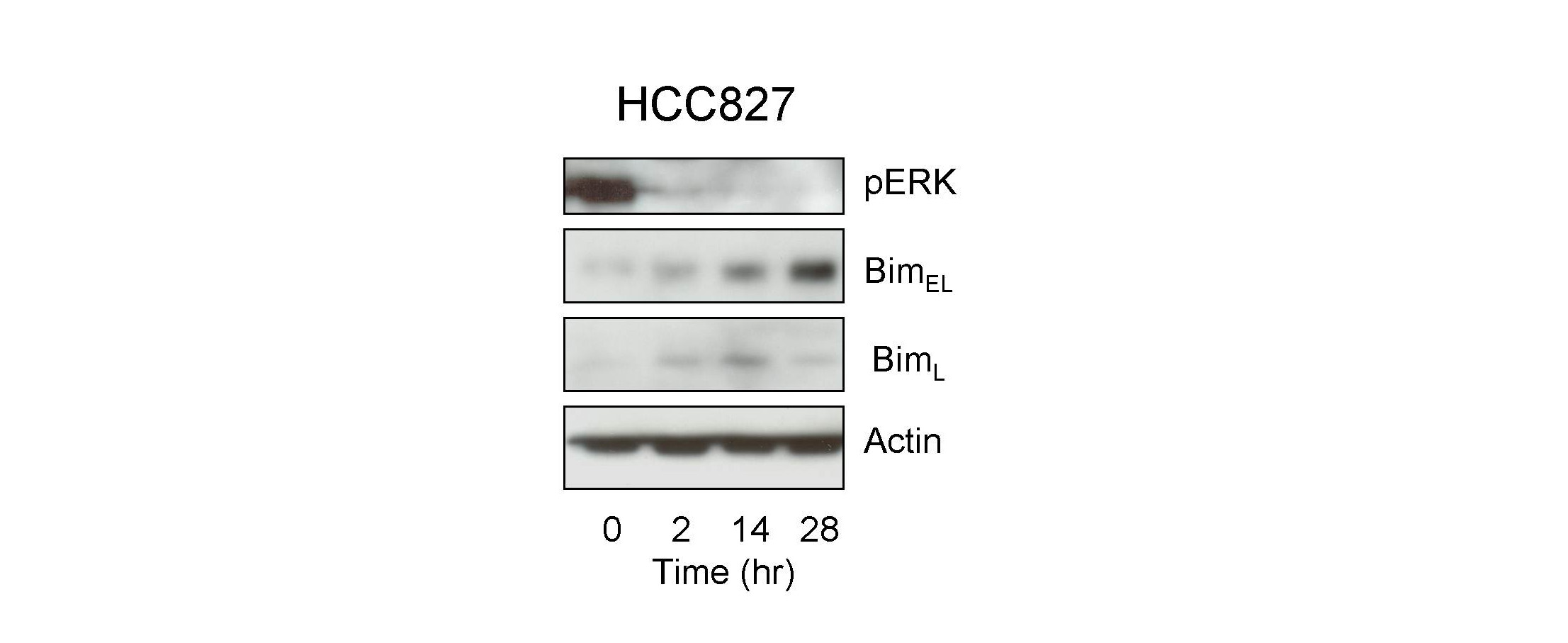

Supplement: Figure S3 — Bim is induced after gefitinib treatment in HCC827 cells and is coincident with ERK dephosphorylation. HCC827 cells were treated with 1 μM gefitinib for 2–28 h and the cells harvested, lysed and assessed by Western blotting for expression of BIM, pERK, and actin (loading control). (119 KB JPG) [file pmed.0040316.sg003.jpg]

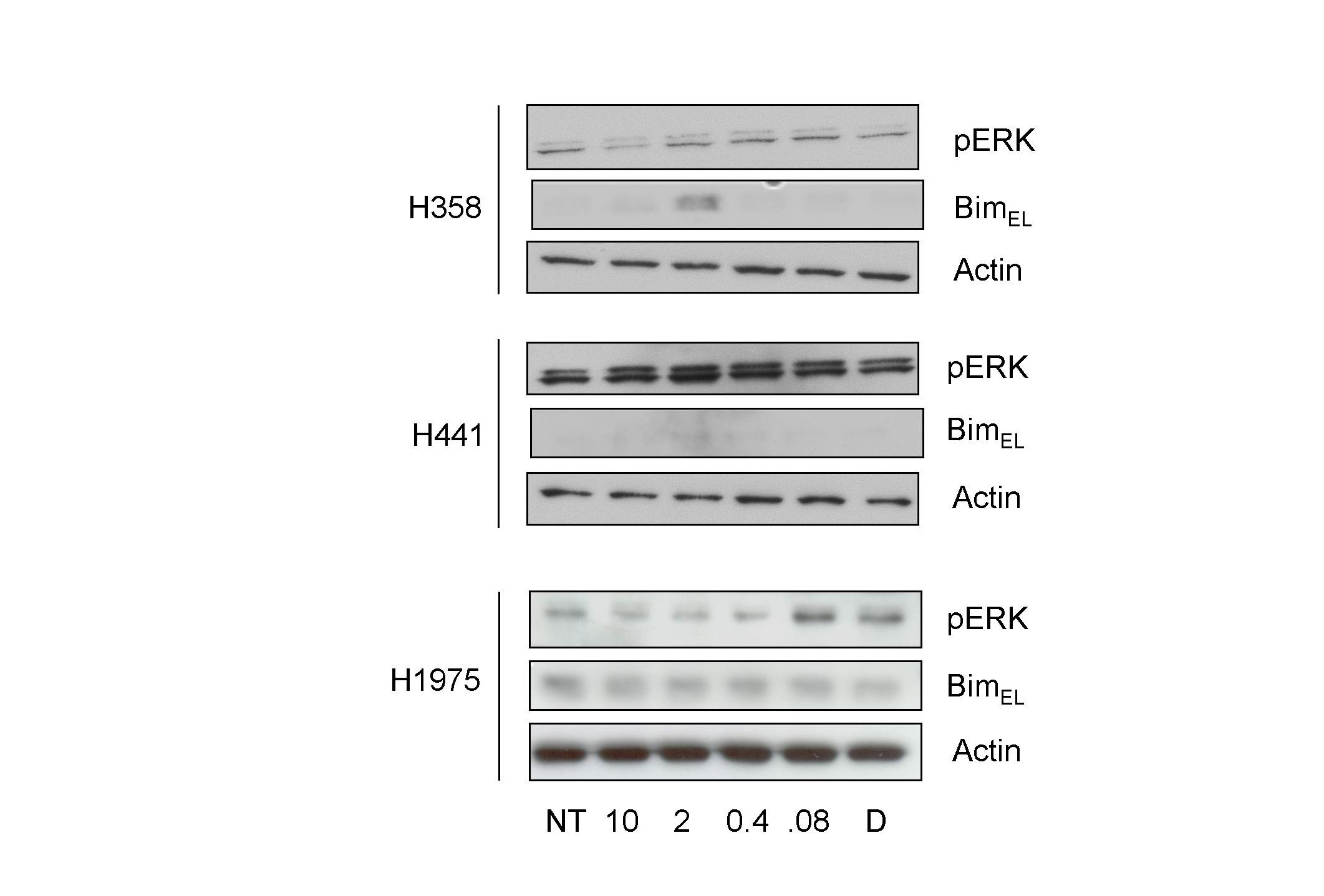

Supplement: Figure S4 — H358 and H441 cells expressing WT EGFR or H1975 cells expressing L858R and T790M mutant EGFR were left untreated (NT) or treated for 24 h with gefitinib (10, 2, 0.4, 0.08 μM) or DMSO (D). The cells were then assessed by Western blotting for the phosphorylation status of ERK1/2 and the level of BIM and actin (loading control). (234 KB JPG) [file pmed.0040316.sg004.jpg]

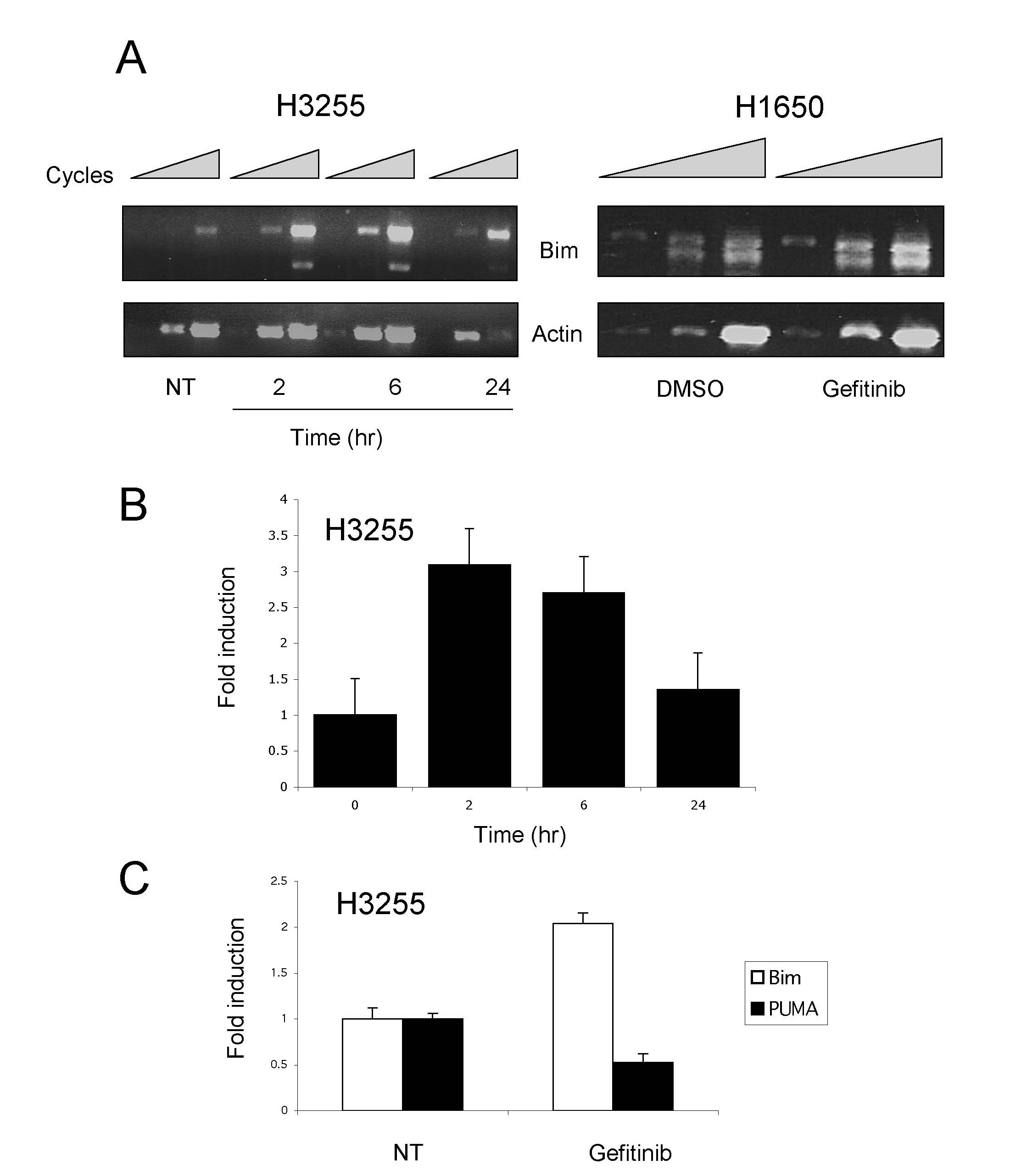

Supplement: Figure S5 — NSCLC cells were treated with 1 μM gefitinib for 2, 6, or 24 h (H3255) or 24 h only (H1650). The cells were then harvested, total RNA isolated and converted to cDNA. Semiquantitative PCR (A) or quantitative PCR (B and C) analysis was then performed to determine the levels of BIM or PUMA. Bars represent the mean ± standard deviation of three independent experiments. (410 KB JPG) [file pmed.0040316.sg005.jpg]

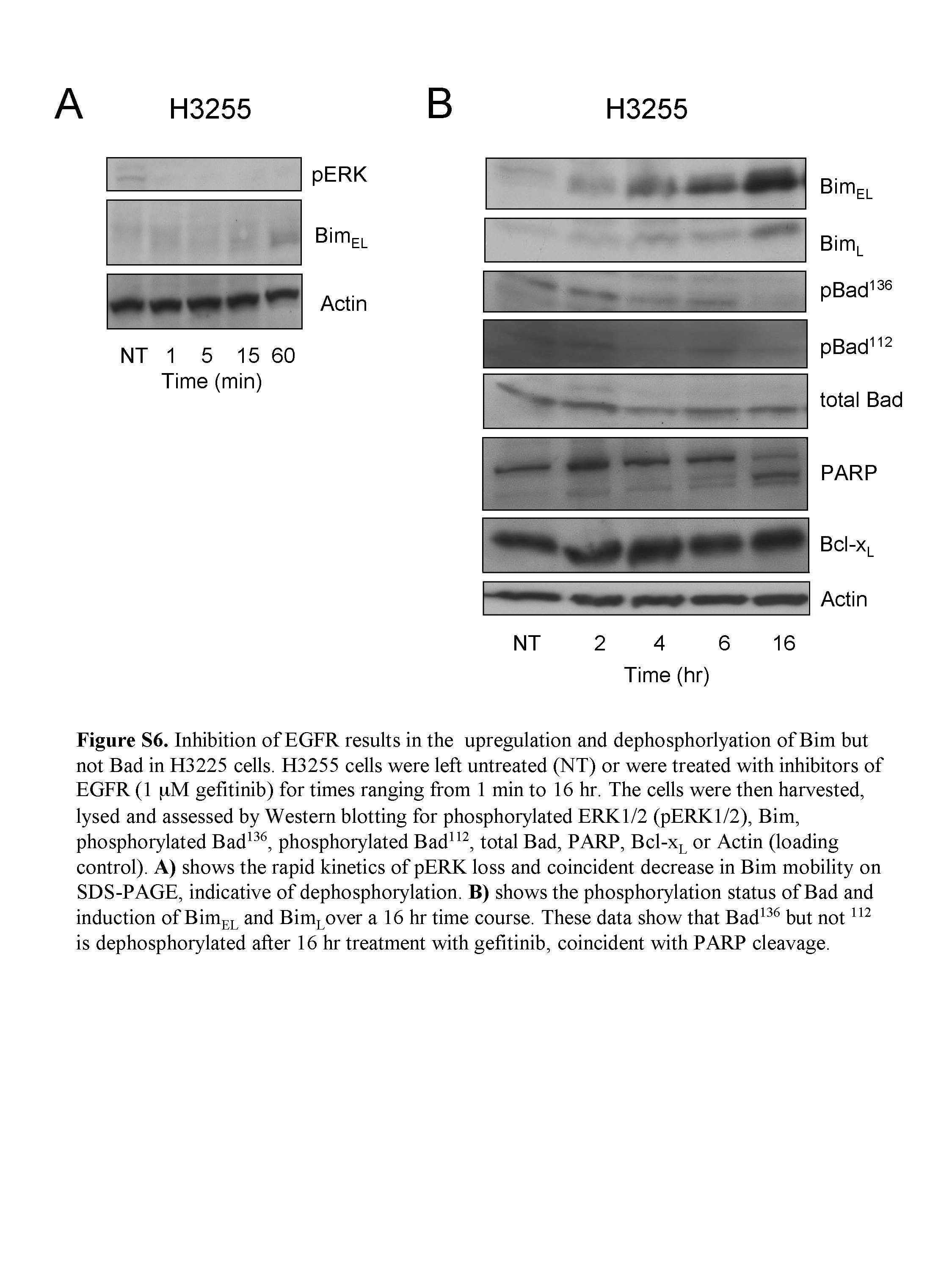

Supplement: Figure S6 — H3255 cells were left untreated (NT) or were treated with inhibitors of EGFR (1 μM gefitinib) for times ranging from 1 min to 16 h. The cells were then harvested, lysed and assessed by Western blotting for phosphorylated ERK1/2 (pERK1/2), BIM, phosphorylated BAD136, phosphorylated BAD112, total BAD, PARP, BCL-xL, or actin (loading control). (A) The rapid kinetics of pERK loss and coincident decrease in BIM mobility on SDS-PAGE, indicative of dephosphorylation. (B) The phosphorylation status of BAD and induction of BIMEL and BIML over a 16 h time course. These data show that BAD136 but not BAD112 is dephosphorylated after 16 h treatment with gefitinib, coincident with PARP cleavage. (379 KB JPG) [file pmed.0040316.sg006.jpg]
